# Conjugation of Carbon Dots with β-Galactosidase Enzyme: Surface Chemistry and Use in Biosensing

**DOI:** 10.3390/molecules24183275

**Published:** 2019-09-09

**Authors:** Shiv K. Sharma, Miodrag Micic, Shanghao Li, Benjamin Hoar, Suraj Paudyal, Elsayed M. Zahran, Roger M. Leblanc

**Affiliations:** 1 Department of Chemistry, University of Miami, 1301 Memorial Drive, Coral Gables, Miami, FL 33146, USA (S.K.S.) (S.P.); 2 MP Biomedicals LLC, 3 Hutton Center, Santa Ana, CA 92707, USA (M.M.) (S.L.); 3 Department of Engineering Design Technology, Cerritos College, 11110 Alondra Boulevard, Norwalk, CA 90650, USA; 4 Department of Chemistry and Biochemistry, University of California, Los Angeles, CA 90095, USA; 5 Department of Chemistry, Ball State University, Muncie, IN 47306, USA

**Keywords:** β-galactosidase, carbon dots, biosensor, Langmuir monolayer, conjugation, lactose

## Abstract

Nanoparticles have been conjugated to biological systems for numerous applications such as self-assembly, sensing, imaging, and therapy. Development of more reliable and robust biosensors that exhibit high response rate, increased detection limit, and enhanced useful lifetime is in high demand. We have developed a sensing platform by the conjugation of β-galactosidase, a crucial enzyme, with lab-synthesized gel-like carbon dots (CDs) which have high luminescence, photostability, and easy surface functionalization. We found that the conjugated enzyme exhibited higher stability towards temperature and pH changes in comparison to the native enzyme. This enriched property of the enzyme was distinctly used to develop a stable, reliable, robust biosensor. The detection limit of the biosensor was found to be 2.9 × 10^−4^ M, whereas its sensitivity was 0.81 µA·mmol^−1^·cm^−2^. Further, we used the Langmuir monolayer technique to understand the surface properties of the conjugated enzyme. It was found that the conjugate was highly stable at the air/subphase interface which additionally reinforces the suitability of the use of the conjugated enzyme for the biosensing applications.

## 1. Introduction

Different nanoparticles and quantum dots have been employed in many applications like catalysis [1,2], imaging [3,4], drug delivery [5,6,7,8], and biosensing [9,10,11]. But their sustainable use has been limited due to their toxicity [12] and other harmful health effects [13], like pulmonary toxicity, translocation to extrapulmonary sites, and eluding phagocytosis. In the other hand, there is increased use of enzymes in food industries [14], pharmaceutics [15] and in the energy industry [16] due to their specific catalytic properties and selectivity. Nonetheless, enzymes have low operational stability and relatively poor reusability. This drawback is linked to their sensitivity to temperature and pH. Different immobilization techniques have been employed to maintain enzyme stability and reusability [17,18]. In this case, the use of inorganic and organic supports can sufficiently change the chemical and physical properties of the enzyme which in turn can decrease its activity. Furthermore, the enzyme when physically adsorbed through weak hydrogen bonds, van der Waals forces, and electrostatic force has a high chance of leaching out easily when the pH of the medium is changed [19].

**Figure 1:**
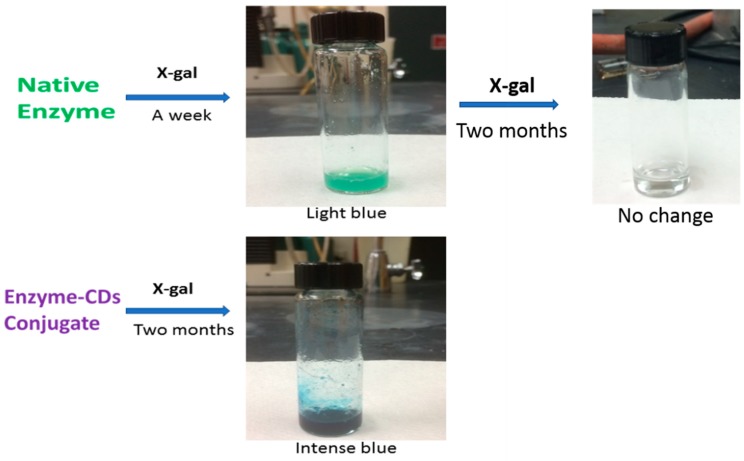
The different changes in color after treating the X-gal with the native and conjugated enzyme stored in room temperature after a week and two months, respectively.

Although the use of carbon nanotubes, several metal nanoparticles, and polymers in biosensing has become quite common [20,21], limited work has been done in incorporating carbon dots (CDs) onto the enzymes. Very recently, CDs (nontoxic nanoparticles) were incorporated onto protein molecules to achieve unique properties. For example, Liu and co-workers have shown that the conjugation of carbon dots with the toxin ricin enhanced its immunomodulatory activity [22]. Immobilization of horseradish peroxidase with carbon dots/CoFe layered double hydroxides has been used in hydrogen peroxide sensing [23]. Similarly, Pradhan et al. have shown that iron oxide nanoparticles conjugated with glutamine- and proline-based osmolytes inhibit protein aggregation [24]. Hosseinzadeh et al. showed that the interaction of quantum dots (QDs) and proteins strongly influenced the surface characteristics of the QDs at the protein-QD interface [25]. A recent review describes the biosensing ability of natural receptor-conjugated conducting nanomaterials [26]. With this perspective in mind, we conjugated non-toxic gel-like carbon dots synthesized in our lab [27] with the enzyme β-galactosidase. Surprisingly, we found that the stability of the enzyme was increased appreciably (Figure 1). Likewise, the enzyme activity did not diminish after storage of the enzyme at room temperature for more than two months. This new property of the enzyme conjugate led to the idea to prepare a biosensor.

In the development of a highly sensitive and advanced biosensor, there are several challenges that limit the detailed mechanism describing the behavior of nanoparticles used, enhancement of signal to noise ratio, transduction, and amplification of signals. Although, metal nanoparticles have been widely used to achieve lower detection limits, they were mostly toxic and had high variations in the analyte detection during the course of batch measurements due to the small variations in the density of the metal nanoparticles [28]. Previously, numerous nanomaterials have been applied as matrices for the immobilization of β-galactosidase in biosensors by several approaches like cross-linking, physical adsorption, and covalent entrapment [29], but they are susceptible to high loss of enzyme during the sensing process. Hence, we designed a method of conjugation of β-galactosidase with CDs in presence of a cross-linking agent, 1-ethyl-3-(3-dimethylaminopropyl) carbodiimide (EDC) to improve the overall sensing capability of the biosensor.

In this paper, we first studied the interfacial behavior of the conjugated enzyme. This study showed that the conjugate was stable enough to remain as a Langmuir monolayer at the air/subphase interface. This module is directly related as the capability of the conjugate to stay active on the electrode surface of the biosensor. Later, an electrochemical biosensor was developed by adsorbing the conjugate on a glassy carbon electrode. The detection limit of the biosensor was found to be 2.9 × 10^−4^ M and its sensitivity was 0.81 µA·mmol^−1^·cm^−2^.

## 2. Results and Discussion

### 2.1. UV-Vis Spectra of the Conjugate

**Figure 2:**
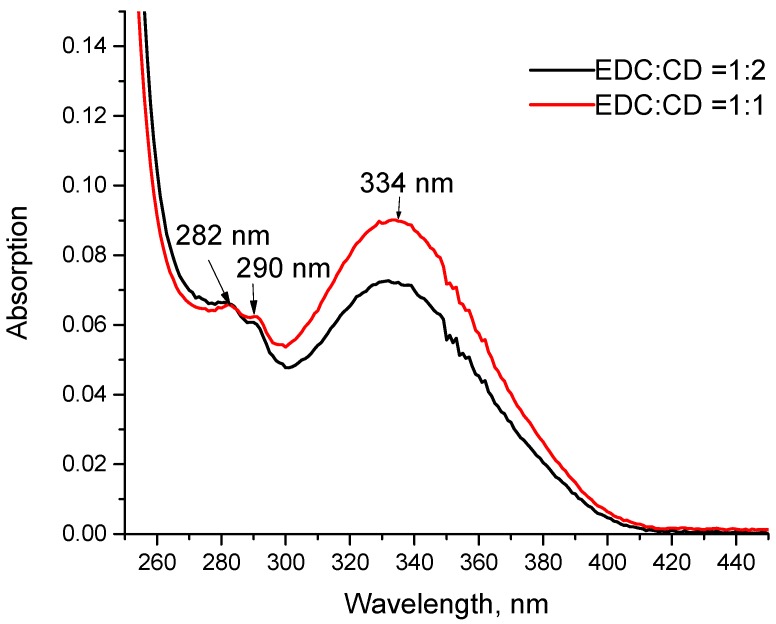
Absorption spectra of the conjugate. Here, the concentration of EDC and CDs was the same in both cases.

The absorption spectra of the β-galactosidase/CDs conjugate are shown in Figure 2. We can see three different peaks at 282, 290, and 334 nm. The first two peaks are due to π–π* transitions and the last one due to n–π* transitions, respectively. The absorption peak for the enzyme is at 280 nm and for the carbon dots it is at 332 nm. The conjugate shows the formation of new bond at 290 which is ascribed to the π–π* transition of a newly amide bond formed. We are not clear about the cause of the suppression of the band at 334 nm when the molar ratio of EDC and CDs was 1:2 as compared to the 1:1 ratio. Here, in both cases the amount of enzyme remains the same. We believe that a 1:1 molar ratio of EDC:CDs is best for the conjugation as the limited amine groups of the enzyme can only bind with the activated CDs.

**Figure 3:**
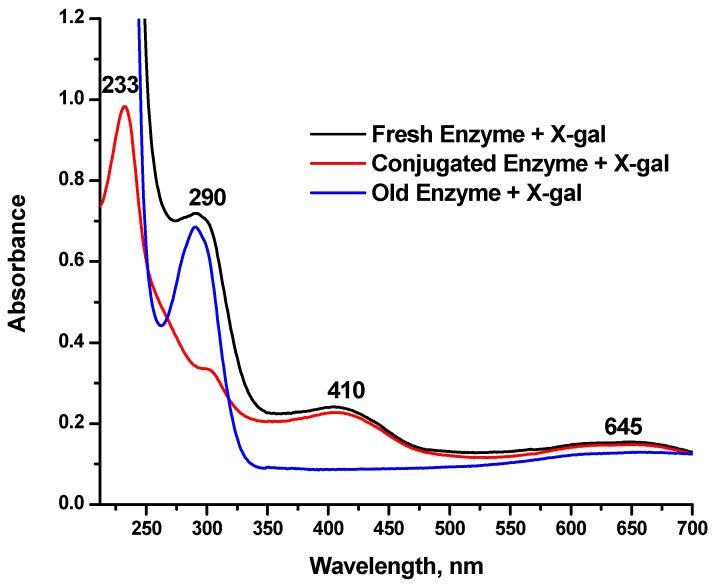
Absorption spectra obtained after treatment of X-gal with fresh, conjugated and old β-galactosidase enzyme.

After obtaining the absorption spectra of the enzyme conjugate, we were interested in knowing the absorption pattern of the fresh, old and conjugated enzyme. Figure 3 clearly shows the differences in the absorptions of these three different states of the enzyme with X-gal. The absorption spectra of conjugated enzyme has four bands at 233, 290, 410, and 645 nm. The band at 410 nm is due to the blue organic moiety (a dimer of 5-bromo-4-chloro-1-indol-3-ol) formed after the cleavage of X-gal by the enzyme. The weak band at 290 nm for the conjugated enzyme is due to the newly formed amide bond. We could not assign the peaks at 233 and 645 nm. Old enzyme does not have the peak at 410 nm. This means that it could not catalyze the X-gal substrate, but it has a sharp peak at 290 nm, which means that although the enzyme′s structure is denatured, the tryptophan (amino acid) molecule is not degraded and has a predominant peak, although it is red-shifted. The absorbance peak of fresh enzyme with X-gal shows the predominant peak at 410 nm similar to the conjugated enzyme which depicts that these states of enzyme were active in catalyzing the breakdown of X-gal substrate.

### 2.2. Fluorescence Spectra of the Conjugate

**Figure 4:**
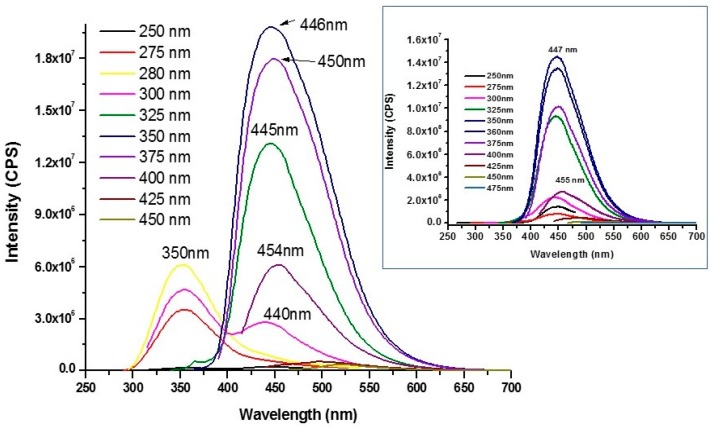
Fluorescence spectra of β-galactosidase-carbon dots conjugate and photoluminescence of G-CDs alone (in the inset).

To further observe the formation of conjugate, we recorded the emission spectra of the conjugate at different excitation wavelength ranging from 250 to 450 nm. The emission at different wavelengths made it easier to compare the emissions of the gel-like carbon dots (G-CDS) and the conjugate as we know that the maximum emission of the enzyme β-galactosidase is at 350 nm (due to the abundance of tryptophan) and the photoluminescence of the G-CDs is excitation wavelength-dependent, which has been already reported in our previous paper [27]. Although the emission spectra of the conjugate are also excitation wavelength dependent, they are completely different as compared to the emission spectra of G-CDs (Figure 4). The emergence of new peaks corresponds to the formation of the conjugate.

**Figure 5:**
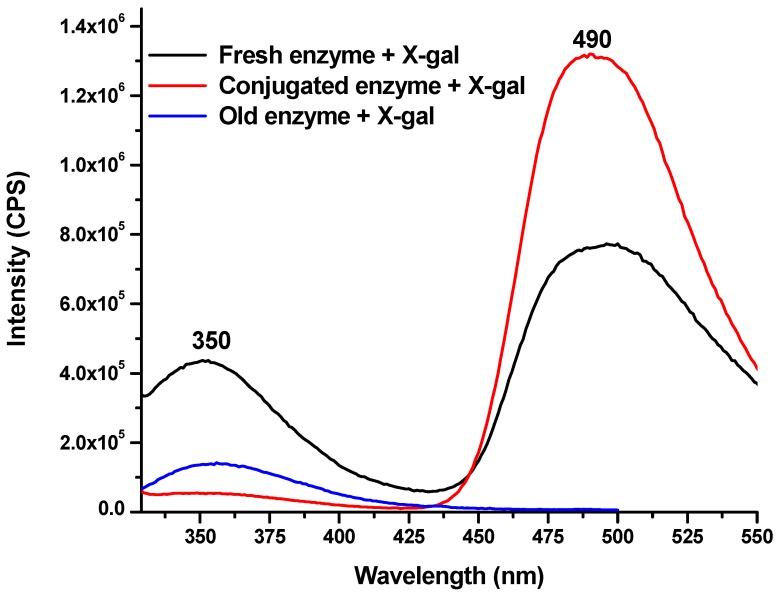
Fluorescence spectra obtained after the catalysis of X-gal by fresh, conjugated, and old β-galactosidase enzyme (excitation wavelength = 290 nm).

Figure 5 shows the fluorescence spectra obtained after the treatment of X-gal by the fresh, conjugated and old β-galactosidase enzyme at the excitation wavelength of 290 nm. The excitation wavelength of 290 nm was chosen because the absorbance maxima was obtained at this wavelength. Mainly two bands were observed at 350 nm and 490 nm. The emission at 350 nm corresponds to the emission of tryptophan, abundant in the enzyme, whereas the emission at 490 nm corresponds to the blue organic moiety formed after the cleavage of X-gal. From the figure, it is clear that old enzyme could not catalyze the X-gal (absence of 490 nm band) whereas fresh and conjugated enzyme were able to catalyze the X-gal. Fresh and old enzyme both have the emission band at 350 nm but this band is really suppressed in the case of conjugated enzyme. This may be due to the presence of highly fluorescent CDs in the conjugated enzyme that could suppress the enzyme emission.

### 2.3. Fourier Transform Infrared (FTIR) Spectroscopy

**Figure 6:**
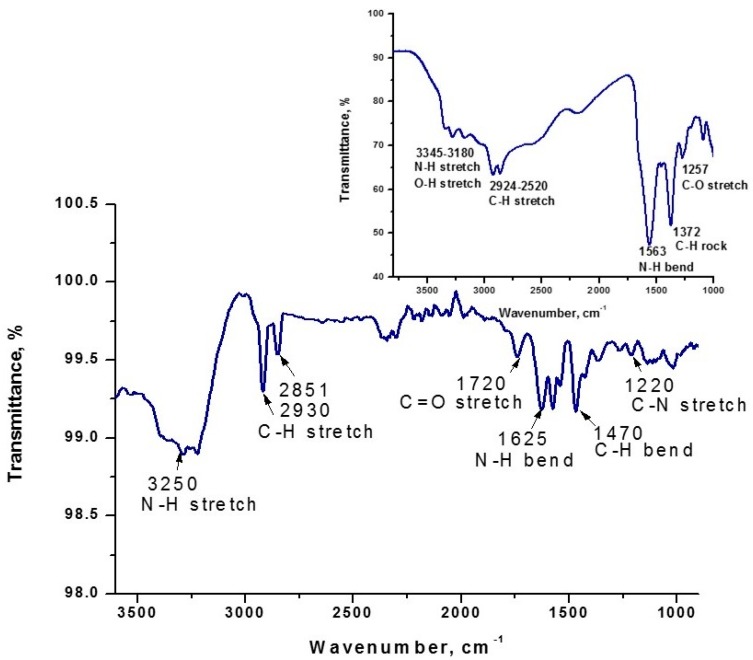
FTIR spectra of β-galactosidase-CDs conjugate (FTIR spectra of bare CDs in the inset).

From the absorbance and fluorescence spectra the formation of amide bonds in the conjugate was not clear. Hence, FTIR spectra were taken to confirm the conjugate formation. Figure 6 shows the FTIR spectra of the conjugate and the bare CDs for comparison. The amide functional group combines the features of amines and ketones as it has both an N–H bond and a C=O bond. As per this combination amides show a very strong, somewhat broad band in the range between 3100 and 3500 cm^−1^, as shown in the figure. This corresponds to the N–H stretch. At the same time, a band positioned at 1720 cm^−1^ resembles the C=O stretch. The other bands at 1625, 1470, and 1220 cm^−1^ correspond to the N–H bend, C–H bend and C–N stretch, respectively. The bands at 2930 and 2851 cm^−1^ are due to C–H stretching.

### 2.4. Study of the Conjugate Cytotoxicity on Sea Urchin Embryos

When a biosensor is dipped in the substrate solution to evaluate analyte concentration, we should be aware that the analyte released, for any reason, is not toxic and does not damage the substrate solution. Practically, the chance of physically adsorbed analyte on the surface of biosensor is very low. To evaluate the toxicity of enzyme-CDs conjugate, we used sea urchin embryos to test for static acute toxicity. The reason for choosing sea urchin embryos is that they are highly sensitive to toxic chemicals and conducive for experimental observations. Initially, gametes were collected from adult sea urchins with ripe gonads. Fresh eggs were washed multiple times with cold seawater and mixed with sperm to examine fertilization rates. Only eggs with a fertilization rate higher than 95% were used for the toxicity tests. Toxicity tests were performed in a clean, 24-well cell culture plate. In each well, 100 healthy fertilized eggs were incubated in 2 mL of seawater with conjugate at a concentration of 0, 5, 10, 20, 50, and 100 μg/mL, respectively. The plate of fertilized eggs was incubated at 15 °C for 16 h until they reached the mesenchyme blastula-stage embryos.

**Figure 7:**
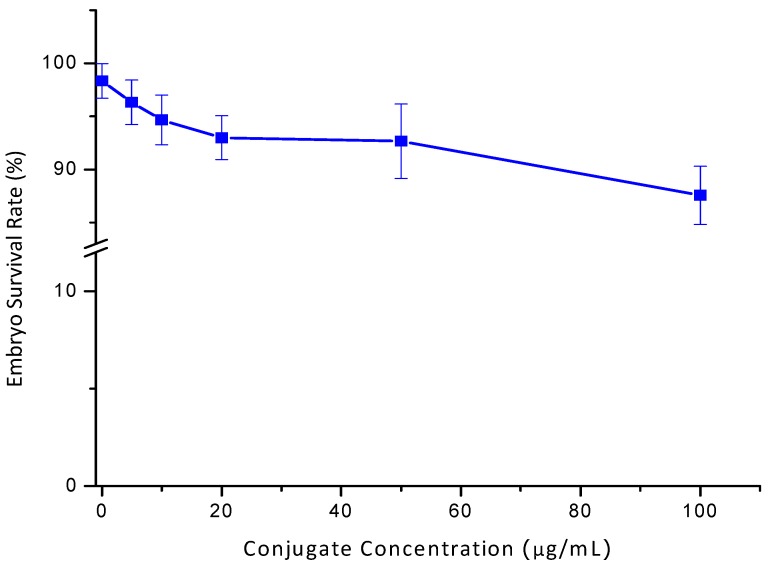
Cytotoxicity study of the conjugate.

The results (Figure 7) show that enzyme conjugate has low cytotoxicity to fertilized sea urchin eggs and embryos. In the presence of 10 μg/mL conjugate, more than 95% of sea urchin embryos retain a normal morphology after 16 h of incubation. This data is similar to when embryos are treated with bare CDs. Even at the highest tested concentration of conjugate (50 μg/mL), more than 90% of embryos remain normal, indicating low cytotoxicity of the conjugate to the cells. This shows that if by any mistake, the conjugate leaks into the substrate solution it wouldn’t have any alarming effects. Low cytotoxicity of the conjugate also shows the potential of using it in other fields like immunoassays too.

### 2.5. Surface Chemistry and Spectroscopic Studies of the Conjugate

#### 2.5.1. Surface Pressure and Surface Potential

We were also interested to know the surface chemistry of the conjugate as its surface properties determine its adsorption behavior on the surface of an electrode [30]. Surface chemistry and spectroscopic studies can also be used to reveal the interaction of the analytes at the air/subphase interface and in bulk solution. 

**Figure 8:**
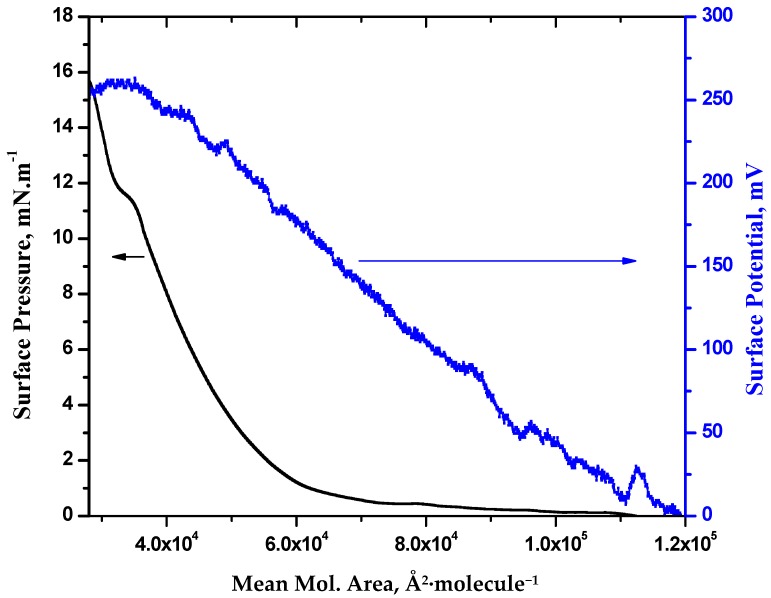
Surface pressure and surface potential isotherm of β-galactosidase-CDs Langmuir monolayer (spreading volume = 45 µL, at room temperature).

Figure 8 shows that the conjugate forms good π–A as well as the surface potential isotherm on the 0.1 M NaCl subphase. The limiting molecular area was determined to be 35,000 Å^2^·molecule^−1^ which describes the cross-sectional area per molecule of the conjugate. The surface potential explains the dipole moment of the analyte below and above the air/subphase interface. Initially, there was sudden increase in surface potential. This is due to the sudden movement of molecules during compression. The crest and troughs seen in the surface potential are due to the rapid movement of conjugate molecules to attain a specific orientation. Finally, there is sudden drop in the surface potential at the collapse surface pressure. This is due to the cancellation of the dipole moment as conjugate molecules come very near to each other.

#### 2.5.2. X-gal Concentration Effects on the Langmuir Monolayer of β-galactosidase-CDs Conjugate 

**Figure 9:**
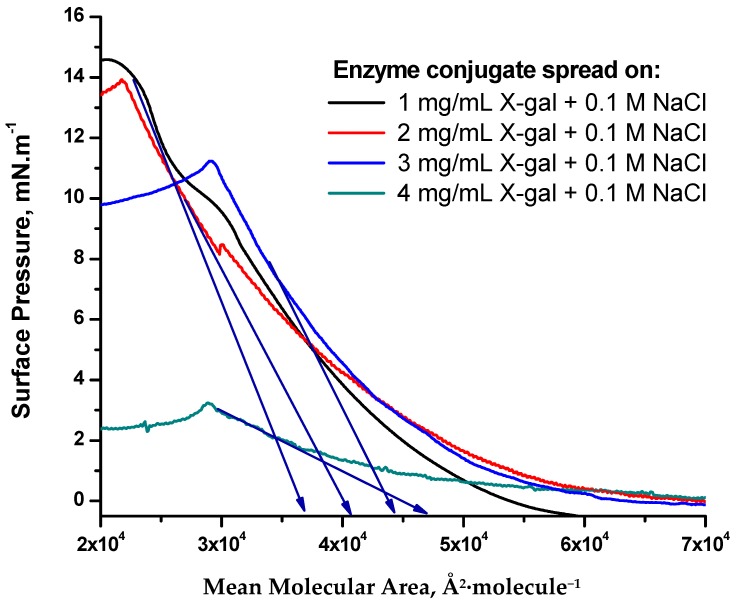
Concentration effect of X-gal on the Langmuir monolayer isotherms of β-galactosidase-CDs conjugate at a concentration of 0.06 mg·mL^−1^.

As X-gal is the substrate of β-galactosidase enzyme, it should certainly have some effects on the Langmuir monolayer of the β-galactosidase-CDs conjugate. As per our expectation, there was change in the Langmuir monolayer of the conjugate with different concentrations of X-gal. Figure 9 provides details of the impact of the different concentrations of X-gal on the β-galactosidase-CDs conjugate Langmuir monolayer. In this experiment 45 µL of conjugate having concentration of 0.06 mg·mL^−1^ was spread on the subphase of X-gal and 0.1 M NaCl by using a fine syringe. We found that with the increase in the concentration of X-gal on the subphase there was increase in the limiting molecular area. The limiting molecular area is associated with the minimum cross-sectional area per molecule. The abovementioned finding can be explained with relation to the interaction of conjugate with the X-gal. We observed significant increase in the limiting molecular area when 4 mg·mL^−1^ of X-gal was present in the subphase. This may be due to the solubilization of conjugate molecules in the subphase because of low ionic strength.

#### 2.5.3. In Situ UV-Vis Spectrum of the Langmuir Monolayer

**Figure 10:**
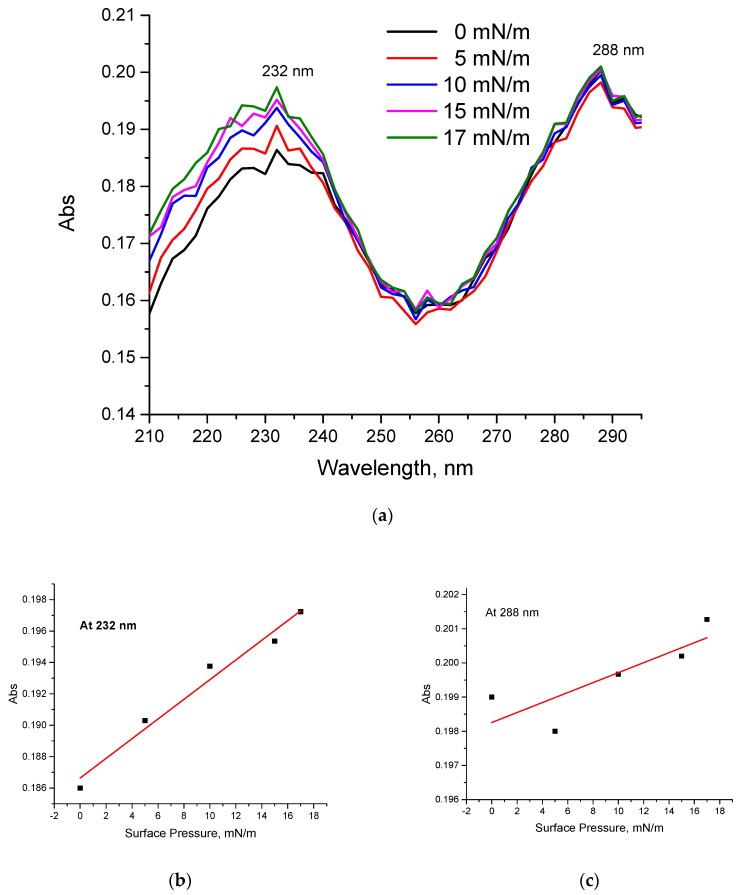
In-situ UV-vis spectra of the enzyme conjugate (0.1 mg/mL) on the subphase of 0.1 M NaCl (**a**). The absorbance intensity of the bands at 232 and 288 nm in is plotted against surface pressure in (**b**) and (**c**), respectively.

After knowing that the formation of conjugate was successful through the techniques like UV-vis, and fluorescence, we were interested in knowing the amount of the conjugate that remains on the interface. This is because it directly relates to the stability of the analyte (conjugate) on the interface of an electrode while constructing the biosensor. As shown in the Figure 10, the band peak at 232 nm can be designated as the combined higher-energy maxima of tyrosine and tryptophan residues obtained from the protein molecule [31]. In this case, the peaks from the induced effect CDs on the conjugate in monolayer were not predominant as like the absorbance obtained for the solution. This can be explained in such a way that the free residual amount of CDs were desorbed in the subphase as the CDs are highly hydrophilic. Furthermore, CDs bonded with enzyme in the conjugate monolayer also remained immersed on the subphase as only hydrophobic component of the analyte remains projected in the air at the air/subphase interface [32].

We plotted the absorbance data obtained for different surface pressures. In doing so a linear relationship was established (Figure 10). This observation reinforces the interpretation that the conjugate of CDs and β-galactosidase remains at the air/subphase interface facilitating the development of a biosensor.

### 2.6. Monitoring the Reaction of the Conjugate and Lactose

**Figure 11:**
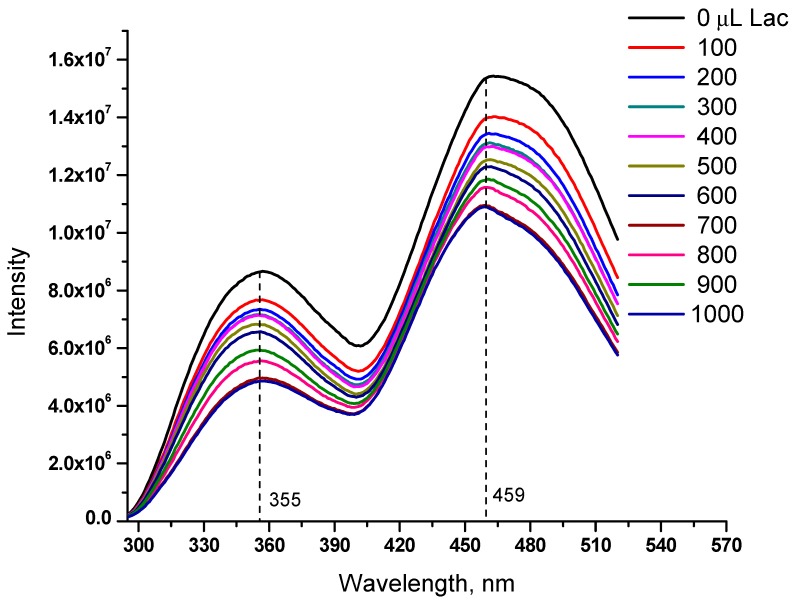
Fluorescence spectra of the conjugate with different concentration of lactose (Exc. λ = 280 nm, path length = 1 cm).

While treating different concentrations of lactose with the conjugate of β-galactosidase/CDs we observed a quenching of the intensity of the emission peaks as shown in Figure 11. We observed this quenching phenomenon with the increasing the lactose concentration.

The quenching of emission usually occurs when the enzyme sites are blocked. We were doubtful that this would impact the electrochemical behavior of the designed biosensor.

#### 2.6.1. Development of a Biosensing Platform

Traditionally, the lactose concentration in milk (4.5–5% of milk (by weight)) is determined by the use of lactase, which splits lactose into glucose and galactose. The glucose reacts with a phenolic compound through an enzymatic reaction, catalyzed by peroxidase, and forms a pink colored complex. The absorbance of the complex is read at 505 nm, and the value is directly proportional to the concentration of lactose in the sample. To avoid the drawbacks of traditional methods and enhance the stability of the enzyme, we conjugated the enzyme with CDs and constructed a working electrode (glassy carbon electrode) by immobilizing the conjugate along with polyethylene glycol (PEG) and activated carbon and used cyclic voltammetry to quantify lactose.

#### 2.6.2. Cyclic Voltammetry Study

We employed cyclic voltammetry as it is the most versatile technique employed for obtaining qualitative information about electrochemical reactions [33]. Besides, it renders the rapid identification of redox potentials distinctive to the electroactive species under investigation, providing considerable information about the thermodynamics of a redox process, kinetics of heterogeneous electron-transfer reactions, and analysis of coupled electrochemical reactions or adsorption processes.

In the experiment, we swept a voltage (−1.0 V to +1.0 V) at a scan rate of 0.1 V/s for a selected range, but we did not see a prominent peak, i.e., no reduction potential existed within that range (curve not shown). This means that there was no redox reaction occurring. The observed data only shows the interaction of conjugate and lactose which later leads to the cleavage of lactose into glucose and galactose.

**Figure 12:**
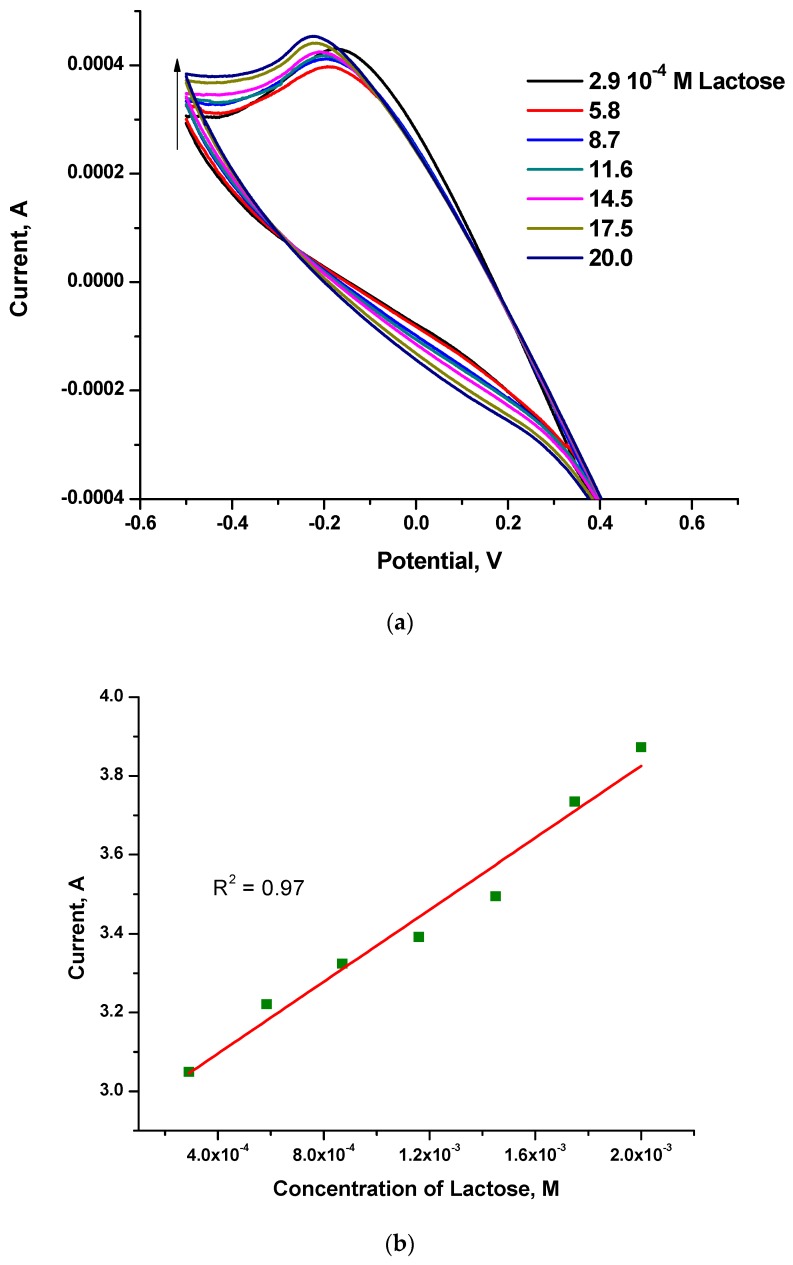
Cyclic voltammogram of the glassy carbon electrode (adsorbed with β-galactosidase/carbon dots conjugate) with different concentrations of lactose in Fe^2+^ solution (**a**) and a plot of the concentration of lactose versus current (**b**).

Adding a redox-active label (Fe^3+^/Fe^2+^) allowed us to monitor the electrode interfacial activity. As the lactose binds to the enzyme, the electrochemical environment of the electrode surface changes as we see from the current signal. Figure 12 illustrates the voltammetric behavior of the conjugate electrode system in hexacyanoferrate solution (1 mM [Fe(CN)_6_]^3−^/[Fe(CN)_6_]^4−^ in 0.1 M PBS, pH 7). It showed a well-defined Faradaic response. We found that current kept on increasing with the increased concentration of lactose added into the Fe^3+^/Fe^2+^ system. This information shows that a redox system is necessary in the β-galactosidase-CDs conjugate electrode system to determine the effective concentration of lactose in the sample.

**Figure 13:**
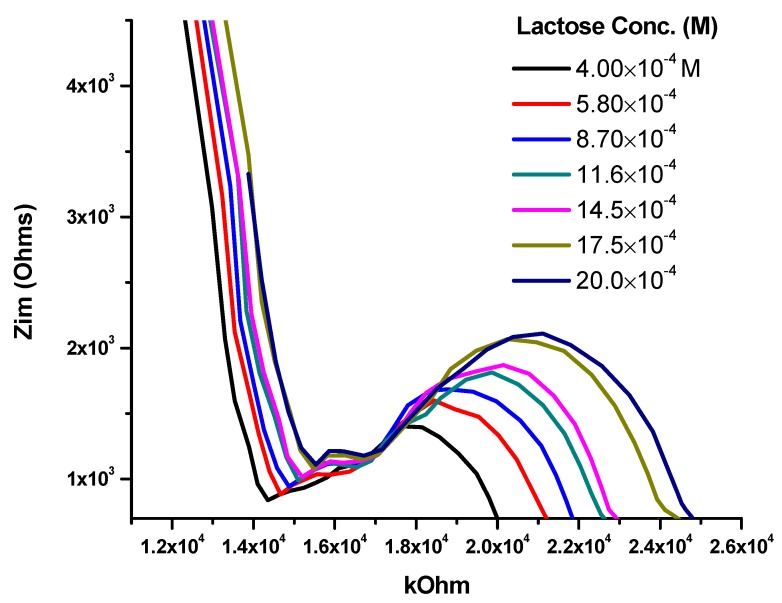
Plot of imaginary (Zim) and resistance, kOhm by using Nyquist plot.

We also used electrochemical impedance spectroscopy to investigate the interactions at the interface of the β-galactosidase-modified electrode upon the addition of lactose. The Nyquist plot in Figure 13 shows a linear portion at the low frequency to indicate the diffusion of the substrate to the electrode surface. In addition, the semicircle part of the plot at high frequencies represents the hindrance of electron transfer limited process because of the binding of lactose to the enzyme. This binding event increases the impedance between the electrode surface and the supporting electrolyte. In this case, the dimeter of the semicircle at higher frequencies represents the corresponding electron transfer resistance (Ret) [34,35].

**Figure 14:**
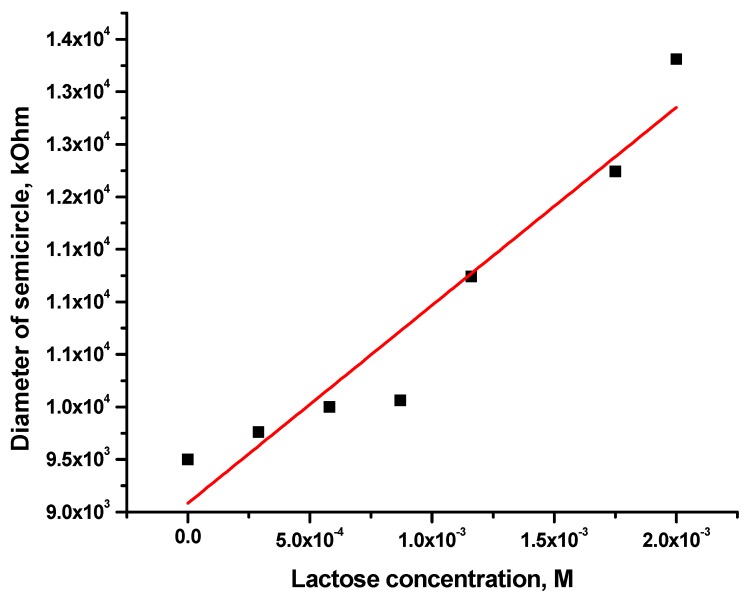
Plot of diameter of the semicircle and concentration of lactose.

Figure 14 indicates gradual increase of the Ret as the concentration of lactose increases, with a linear range of 2.9 × 10^−4^–20.0 × 10^−4^ M. These results suggest that the β-galactosidase carbon dots conjugate binds lactose at the surface of the electrode, which increases the dielectric layer at the electrode solution interface. The detection limit for the biosensor was 2.9 × 10^−4^ M. The sensitivity of the biosensor was evaluated based on the standard deviation of the plot of current vs concentration of lactose. It was found to be 0.81 µA mmol^−1^·cm^−2^. The impedance data also showed that the byproducts, like glucose and galactose, do not interfere.

## 3. Materials and Methods

### 3.1. Chemicals

β-Galactosidase and the EDC required for the conjugation were purchased from MP Biomedicals (Irvine, CA, USA). Gel-like CDs were synthesized in the lab. For the synthesis of CDs, citric acid (99.5–100%) was purchased from VWR (Radnor, PA, USA), and EDA (99%) and quinine hemisulfate monohydrate from Alfa Aesar (Tewksbury, MA, USA). Hydrochloric acid, Sodium chloride (99%) and sodium hydroxide were obtained from MP Biomedicals (Irvine, CA, USA). Compressed argon gas with ultra-high purity (99.99%) was procured from Airgas (Radnor, PA, USA).

### 3.2. Synthesis of CDs

Initially, EDA (5 mL) was heated in an oil bath up to 160 °C in a round bottom flask and 1 g of citric acid was added to it. The solution was heated constantly for 50 min until the citric acid reacted completely with the EDA. The reaction mixture was allowed to cool for 15 min to room temperature. At this time, the G-CDs start depositing at the bottom of the flask. The supernatant EDA was rinsed out with acetone. Then, CDs were then dissolved in 5 mL of water and heated using a rotavapor to completely evaporate the water at constant temperature under reduced pressure. Ultimately, gel-like CDs are formed, which we have named G-CDs.

### 3.3. Conjugation of β-Galactosidase with CDs

**Scheme 1:**
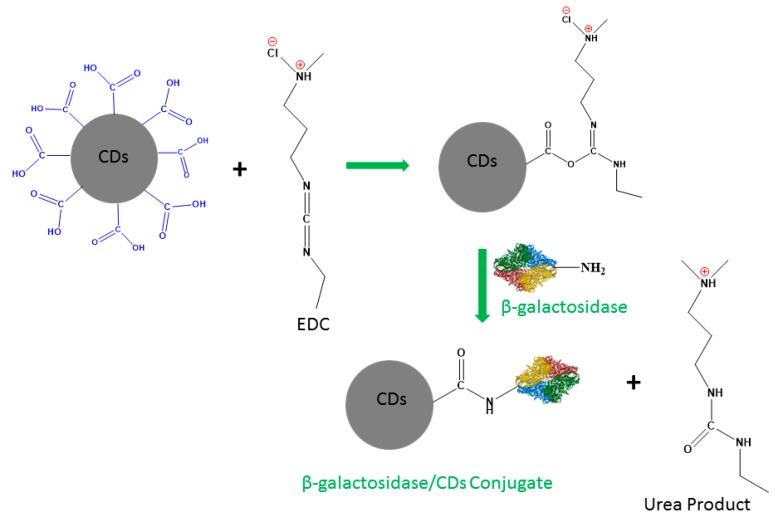
Mechanism of conjugation of β-galactosidase and CDs in presence of EDC.

CDs (2 mg/mL) were treated with EDC and stirred for half an hour. Then freshly prepared β-galactosidase (0.2 mg/mL) was mixed with this solution and stirred for 3 h. The hybrid biocomposite was sonicated and centrifuged at 1500 rpm to remove any loosely bound CDs and β-galactosidase. After sonication and centrifugation, dialysis was performed to remove small unreacted carbon dots. The carefully collected solution was analyzed by UV-vis spectroscopy, fluorescence, and FTIR to check whether the β-galactosidase was strongly bound to the CDs. All the experiments were repeated at least three times to ensure reproducibility. A schematic mechanism for the conjugation process of CDs and β-galactosidase is shown in Scheme 1.

### 3.4. Characterization of the Biocomposite

Characterization of any conjugate is very important for its selective use in biosensing. We used UV-vis, fluorescence, and other in-situ methods to characterize the enzyme/CDs conjugate. Cyclic voltammetry was used to measure the biosensing ability of the CDs/enzyme conjugate.

### 3.5. Immobilization of Conjugate on the Glassy-Carbon Electrode

After characterization of the CDs/β-galactosidase enzyme, it is important to immobilize on the electrode surface to examine its biosensing properties. A glassy-carbon electrode was used to immobilize the conjugate on its surface. Freshly prepared conjugate solution (2 mL) was mixed with carbon powder (100 mg) to form a paste. The paste was gently spread onto the surface of a glassy carbon electrode. After 5 min of drying, the electrodes were ready to carry out cyclic voltammetry experiments. The experiments were repeated at least three times to verify the reproducibility of the results.

## 4. Conclusions

In conclusion, the Langmuir monolayer isotherm of β-galactosidase-CDs conjugate was studied using the surface chemistry and spectroscopy at the air/subphase interphase. The study of the β-galactosidase-CDs conjugate Langmuir monolayer reveals that there is decrease in collapsed surface pressure with the increase in the amount of X-gal in the subphase. Also, it was found that the monolayer is stable. Further, the bulk phase study of the conjugate revealed that it was highly stable, and its activity lasted for at least two months, while the native enzyme only retains its activity for a few days at room temperature. This property of the conjugate was employed in the development of a biosensor. The study of the reaction between conjugate and lactose via fluorescence spectroscopy and cyclic voltammetry showed that interaction that occurs between the conjugate and lactose is sufficient to break the lactose into glucose and galactose. The detection limit of the developed biosensor was 2.9 × 10^−4^ M, whereas its sensitivity was 0.81 µA mmol^−1^·cm^−2^. Moreover, the biosensor was stable for more than two months.
